# *Salmonella* Genomic Island 1 is Broadly Disseminated within Gammaproteobacteriaceae

**DOI:** 10.3390/microorganisms8020161

**Published:** 2020-01-23

**Authors:** Max Laurence Cummins, Mohammad Hamidian, Steven Philip Djordjevic

**Affiliations:** 1 The ithree institute, University of Technology Sydney, Ultimo, NSW 2007, Australia; max.cummins@uts.edu.au (M.L.C.); mohammad.hamidian@uts.edu.au (M.H.); 2 Australian Centre for Genomic Epidemiological Microbiology, University of Technology Sydney, PO Box 123, Broadway, NSW 2007, Australia

**Keywords:** *Salmonella* genomic island 1 (SGI1), antimicrobial resistance (AMR), *Proteus* genomic island (PGI), *Vibrio* genomic island (VGI), *Klebsiella* genomic island (KGI), genomics

## Abstract

*Salmonella* genomic island 1 (SGI1) is an integrative mobilisable element that plays an important role in the capture and spread of multiple drug resistance. To date, SGI1 has been found in clinical isolates of *Salmonella enterica* serovars, *Proteus mirabilis*, *Morganella morganii*, *Acinetobacter baumannii*, *Providencia stuartii*, *Enterobacter spp,* and recently in *Escherichia coli*. SGI1 preferentially targets the 3´-end of *trmE*, a conserved gene found in the Enterobacteriaceae and among members of the Gammaproteobacteria. It is, therefore, hypothesised that SGI1 and SGI1-related elements (SGI1-REs) may have been acquired by diverse bacterial genera. Here, Bitsliced Genomic Signature Indexes (BIGSI) was used to screen the NCBI Sequence Read Archive (SRA) for putative SGI1-REs in Gammaproteobacteria. Novel SGI-REs were identified in diverse genera including *Cronobacter* spp, *Klebsiella* spp, and *Vibrio* spp and in two additional isolates of *Escherichia coli*. An extensively drug-resistant human clonal lineage of *Klebsiella pneumoniae* carrying an SGI1-RE in the United Kingdom and an SGI1-RE that lacks a class 1 integron were also identified. These findings provide insight into the origins of this diverse family of clinically important genomic islands and expand the knowledge of the potential host range of SGI1-REs within the Gammaproteobacteria.

## 1. Introduction

*Salmonella* genomic island 1 (SGI1) is an integrative mobilisable element that carries diverse antibiotic resistance genes, often conferring multidrug resistance [1]. SGI1 was shown to be associated with globally dispersed *Salmonella enterica* serovar Typhimurium DT104 that rose to prominence in the 1980s. Since then, variants of SGI1, including some that carry genes encoding resistance to critically important antimicrobials, have been identified within diverse serovars of *Salmonella enterica* and other Gammaproteobacteria including *Proteus*, *Acinetobacter*, *Morganella*, *Providencia*, *Enterobacter,* and *Escherichia* [2,3,4,5,6]*,* all of which are known human and/or animal pathogens.

While experimental integration of SGI1 into genera such as *Vibrio* and *Klebsiella* has been described [7], there are no detailed reports of an SGI1-related element (SGI1-RE) in wild type strains belonging to these genera. Numerous Gram-negative genera carry the *trmE* gene, the integration site for SGI1 [8], suggesting that SGI1 may be dispersed more broadly in Gammaproteobacteria than is reported in the literature. Here, we aimed to explore the SRA to garner a greater understanding of inter-genera distribution of this clinically important integrative genomic element.

## 2. Materials and Methods

### 2.1. Preliminary Screening of SRA for SGI1-REs and Subsequent Genome Assembly

BIGSI [9] allows for ultra-fast searching of bacterial and viral genomic data for nucleotide sequences of interest, providing access to the 457,000 whole genome sequence (WGS) datasets uploaded to SRA prior to December, 2017. These whole genome sequence datasets were screened for forty-four coding sequences sourced from SGI1 (GenBank accession No. AF261825), constituting all coding sequences from SGI1 other than those downstream of its 3′ end, using the BIGSI web tool (Available online: http://www.bigsi.io/) in combination with a custom-built script available at https://github.com/maxlcummins/SGI1-REs. Retrieved accession numbers and their putative genera classifications, as designated by BIGSI via integrated Kraken [10] and Bracken [11] based analyses, were filtered to remove strains identified as *Salmonella*, *Proteus* and *Acinetobacter*; genera that have been reported previously to carry SGI1-REs. Kraken and Bracken were also used to identify genomes that were contaminated with non-host DNA. Genomic data sets were then downloaded using parallel-fastq-dump version 0.6.3 (Available online: https://github.com/rvalieris/parallel-fastq-dump) and were assembled with Shovill version 1.0.4 (Available online: https://github.com/tseeman/shovill) using default settings.

### 2.2. Putative Identification and Clustering of SGI1-RE Variants

A non-redundant nucleotide database consisting of genes sourced from various SGI1-REs, available at https://github.com/maxlcummins/SGI1-REs, was generated using CD-hit version 4.8.1. Assemblies were screened for the carriage of these genes using BLASTn with a nucleotide identity threshold and coverage threshold of 90%. Strains with identical genotypic profiles, relative to these screened genes, were preliminarily considered to carry the same SGI1-RE. This analysis also informed the particular scaffolds on which genes from the backbones of SGI1-RE were localised.

### 2.3. Annotation and Comparative Analysis of SGI1-REs

For each respective strain found to carry a putative SGI1-RE, BLASTn was used to determine the location of the *trmE* and S044 genes, the former of which contains the 5’ DR-L (terminal 18 bp of the *trmE* gene) and the latter of which is typically seen adjacent to the 3’ DR-R (imperfect direct repeat of DR-R). This region of sequence, inclusive of the DR-R and DR-L, was then isolated and screened against publicly available SGI1 sequences deposited in the NCBI nucleotide collection using MEGABLAST. Following identification of closely related SGI1-RE sequences on NCBI, genome annotation was performed manually using Snapgene (https://www.snapgene.com) version 4.1.9.

### 2.4. Phylogenomic Analysis and Genotypic Characterization of Clonal Subpopulations

Cases where multiple strains of the same predicted genera were found to carry identical SGI1-RE genotypes (see above) were interrogated to determine the phylogenetic and genotypic relatedness of such strains. Phylogenetic analysis was undertaken using Snippy (Available online: https://github.com/tseeman/snippy) and Gubbins [12] as previously described [13]. Phylogenetic trees were generated using FastTree2 version 2.1.10 [14]. Genotyping was undertaken using Abricate (Available online: https://github.com/tseeman/snippy) version 0.9.3. Trees were visualised and annotated using R version 3.6.0 with a custom script available at https://github.com/maxlcummins/SGI1-REs.

### 2.5. Data Availability

Genomic data sets can be retrieved from National Centre for Biotechnology Information’s (NCBI) Sequence Read Archive (SRA) via the following Sequence-Read Archive Accession numbers: *Cronobacter sazakii*: SRR1619558; *Escherichia coli*: ERR1617816, ERR1622475, SRR3987521, SRR4786187; *Klebsiella pneumoniae*: ERR314398, ERR314418, ERR314466, ERR314533, ERR314534, ERR314541, ERR314542, ERR314543, ERR314544, ERR314545, ERR314546, ERR486792*; Vibrio cholerae*: ERR117476, ERR180912, ERR386635, ERR386714, ERR386735, ERR386756, ERR572602, ERR572603, ERR572604, ERR572741, ERR579067, ERR579915.

Please note that we are not the submitters for these specific genomic data sets. Short read sequences utilised in this investigation are all publicly available on the Sequence Read Archive (SRA). For specific SRA accession numbers please see Table S1.

The nucleotide accession numbers pertaining to SGI1-REs identified during this investigation for KGI, PGI2-Ec-2, SGI1-Vc2CHAMA and VGI are MN708012, MN708013, MN708014, and MN708015, respectively.

## 3. Results and Discussion

Thirty strains belonging to the genera *Vibrio* (*n* = 12/30), *Klebsiella* (*n* = 12/30), *Escherichia* (*n* = 5/30) and *Cronobacter* (*n* = 1/30) were found to carry at least one open reading frame associated with the backbone of SGI1 (Table S1). These 30 sequences were identified after Kraken and Bracken analyses were used to exclude contaminated sequences and filtering of samples belonging to genera with multiple reports of SGI1-RE carriage (i.e. *Salmonella*, *Proteus* and *Acinetobacter*). Preliminary BLAST analysis using a non-redundant nucleotide database of SGI1-RE coding sequences indicated that 12 different SGI1-RE variants were present within the 30 isolates (data not shown). Of these, four variants could be localised to a single scaffold, while the remaining seven assembled from multiple scaffolds. Strain metadata and their associated SGI1-RE variants are listed in Table S1.

### 3.1. Potential Discovery of an Ancestral Variant of Salmonella Genomic Island 1

**Figure 1:**
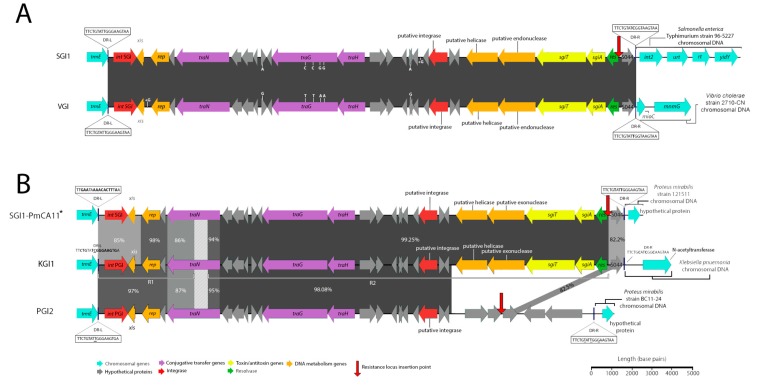
Schematic representation of *Salmonella* genomic island 1-related elements (SGI1-REs) identified in this investigation that are void of integrons. Scale is approximate. Closely related SGI1-REs from public databases are shown atop and below to allow visualization of the nucleotide identity across the novel elements and the references. Nucleotide identities of *trmE* genes (not shown) did not exceed greater than 75% for Vibrio genomic island (VGI) or Klebsiella genomic island (KGI) in comparison with the *trmE* genes of other sequences represented in this schematic. (**A**) A comparison of SGI1 (Accession No: AF261825) shown atop VGI (Accession No. MN708015). Identities and locations of single nucleotide polymorphisms are indicated atop vertical lines at the top and bottom of either element, while nucleotide insertions are indicated in the same fashion but denoted with a plus symbol (+). Genetic context inferred from S. Typhimurium DT104 (GenBank accession number HF937208). (**B**) A comparison of KGI (Accession No. MN708012), PGI2 (Accession No. MG201402) and SGI1-PmCA11 (Accession No. MH990673). Due to the lower nucleotide identity matching between these structures the locations and identities of mismatches and indels are not indicated for ease of interpretation. SGI1-PmCA11*: genetic context inferred from Xiao et al (2019) [16].

A schematic diagram of the SGI1-RE from Vibrio strain 2710-CN (SRA Accession No. ERR579067) that was isolated in China in 1986 is depicted in Figure 1A. Our analysis shows that 2710-CN carries an SGI1-RE sharing 99.97% sequence identity with the backbone of SGI1 (GenBank Accession No. AF261825). To our knowledge, this structure represents the earliest known example of an SGI1-RE. The SGI1 variant, here named VGI (Accession No. MN708015), does not harbour a class 1 integron and, given the extensive sequence identity shared with SGI1, may constitute an ancestral structure from which SGI1 and potentially other SGI1-REs may have evolved.

A recent investigation reported the identification of an SGI1-RE, SGI0 (NCBI Accession No. MG201402.1), which also lacks a class 1 integron [15]. Notably, however, VGI is more closely related to both SGI1 and SGI2 than SGI0. SGI0 and VGI differ by 972 single nucleotide polymorphisms (SNPs), whereas VGI and the SGI1 backbone differ by just 6 SNPs and VGI and SGI2 backbones differ by only 136 nucleotides. It is, therefore, more likely that SGI1 and SGI2 are descendants of a VGI-like element than they are descendants of SGI0.

### 3.2. Identification of a Clonal Outbreak of Extensively Drug-Resistant Klebsiella pneumoniae Carrying a Novel SGI1-RE

*Klebsiella pneumoniae* strain 2485STDY5477984 (SRA Accession No. ERR314534) was found to carry a novel and complex SGI1-RE backbone (Figure 1B). This structure, referred to as KGI (Accession No. MN708012), can be classified into two distinct regions (R1 and R2) based on the SGI1-REs with which it shares extensive sequence identity. Spanning from DR-L to base 5984, R1 is most closely related to PGI2 (Accession No. MG201402). Secondly, R2 constitutes a region from base 6969 to 27,754, corresponding to an intragenic region of S005 and an intergenic region between *res* and S044, respectively. This portion of KGI is most closely related to SGI1-PmCA11 (Accession No. MH990673), with which it shares 99.25% nucleotide identity. It is notable that R1 and R2 are separated by a 985 bp region within S005 which shares low nucleotide identity with the corresponding region in SGI1 and PGI2. Analysis using BLASTn indicates that this region matches part of the *traN* gene of PGI1 (Accession No. KJ411925) with 87% nucleotide identity, despite an alignment of the full *traN*_PGI1_ and *traN*_KGI_ coding sequences revealing a shared nucleotide identity of just 77% and a coverage of just 81%. KGI also features an S044 gene with relatively low nucleotide identity to SGI1 and PGI2 (82% and 83%, respectively), which instead best matches S044_PGI1_—a gene with which it shares 99% sequence identity. This data indicates that KGI likely has a complex evolutionary history and has a mosaic structure resulting from homologous recombination events involving different SGI1-REs.

**Figure 2:**
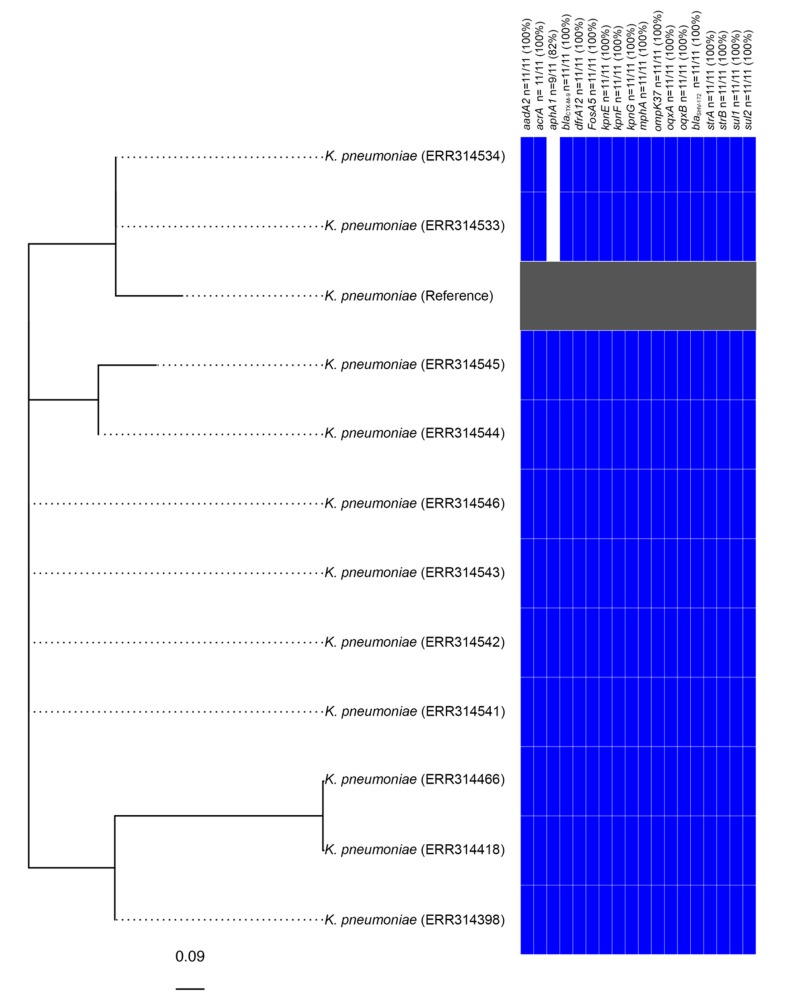
Phylogenetic analysis of *Klebsiella* strains found to carry KGI. The tree is unrooted and the reference strain utilised corresponds to an assembly of *Klebsiella pneumoniae* strain 2485STDY5477984 (SRA Accession No. ERR314534).

It is notable that a further 10 strains from the same Bioproject (PRJEB1271), several of which were listed as being sourced from humans in the United Kingdom [17], also carried an element identical to KGI. Subsequent analysis determined that these strains are also genotypically extensively drug-resistant (XDR), exhibited nearly identical genotypic resistance profiles (two samples did not carry *aphA1*) and differed from one another by between zero and five SNPs (Figure 2). These strains, therefore, appear to constitute a clonal outbreak of an SGI1-RE carrying XDR species that are a frequent cause of nosocomial infections.

### 3.3. Novel Variants and Hosts of SGI1-Related Elements

**Figure 3:**
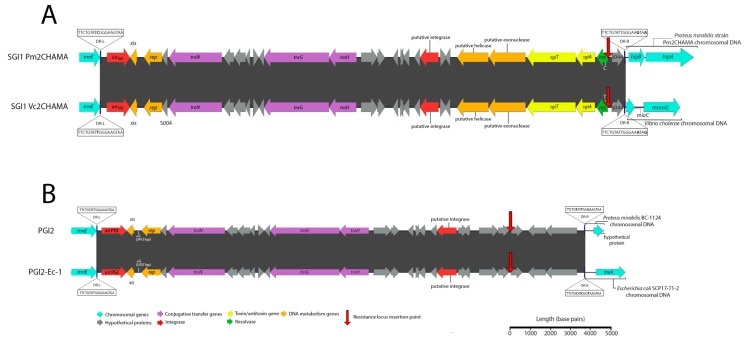
Schematic depicting SGI1-REs identified in this investigation. Scale is approximate. Closely related SGI1-REs from public databases are shown to allow visualization of the nucleotide identity across the novel elements and the associated references. Identities and locations of single nucleotide polymorphisms are indicated atop vertical lines at the top and bottom of either element, while nucleotide insertions are indicated in the same fashion but denoted with a plus symbol (+). Nucleotide identities of *trmE* genes (not shown) did not exceed greater than 75% for SGI1 Pm2CHAMA or PGI2-Ec-1 in comparison with the *trmE* genes of other sequences represented in this schematic. (**A**) A comparison of SGI1 Pm2CHAMA (Accession No: MF372716) shown atop SGI1 Vc2CHAMA (Accession No. MN708014). (**B**) A comparison of PGI2 (Accession No. MG201402) and PGI2-Ec-1 (Accession No. MN708013).

A second SGI1-RE, here named Vc2CHAMA, was identified in *Vibrio cholerae* strain 59-sc-2011-11-25T13:51:24Z-1311470 (SRA Accession No. ERR117476). Vc2CHAMA shares 99.99% sequence identity with SGI1 Pm2CHAMA (Accession No. MF372716.1) and carries a single In*4*-type integron (Figure 3A). Both structures carry a *dfrA15* gene cassette and include a unique 34 bp deletion at the start of S044 caused by IS*6100*—a deletion not otherwise detected by MegaBLAST in the NCBI nucleotide database and one that indicates that SGI1 Pm2CHAMA and Vc2CHAMA share a recent common ancestor. SGI1 Pm2CHAMA and SGI1 Vc2CHAMA differ however in that the former carries a complex resistance region containing two integrons, multiple copies of IS elements, including IS*26*, and an additional five antimicrobial resistance genes (*aadA1, aph1a, strA, strB, and bla_CARB_-4*). It is possible that SGI1 Vc2CHAMA may have arisen from a homologous recombination event between the two integron integrase genes in Pm2CHAMA. Alternatively, Pm2CHAMA arose from a Vc2CHAMA-like structure via homologous recombination event or via an IS*26*-mediated event introducing the upstream integron and other associated elements of the complex resistance locus.

Additionally, a PGI2 variant, here named PGI2-Ec-1 was identified in *Escherichia coli* strain SCP17-71-2 (SRA accession No. ERR1617816). This strain was identified in a study investigating the phylogenetic relatedness of 1,798 ESBL-producing Enterobacteriaeceae from a singly hospital in the Netherlands [18]. PGI2-Ec-1 exhibits 99.28% sequence identity (99% sequence coverage) following BLASTn alignment with PGI2 (Accession No. MG201402) (Figure 3B). PGI2-Ec-2 carries a single In*4* type integron containing *dfrA5*. In contrast, PGI2 carries a complex and mosaic resistance locus featuring two integrons, numerous copies of IS*26* and other insertion sequences. Similarly, with SGI1-Vc2CHAMA, PGI2 and PGI2-Ec-1 could have evolved in either direction via integron integrase associated homologous recombination events or by IS*26*-mediated insertion. While a recent study of ours reported the first identification of an SGI1 variant within *E. coli,* PGI2-Ec-2 constitutes, to our knowledge, the first report of a PGI2 variant within the *Escherichia* genus. This sequence type, ST117, is known to cause extraintestinal infections in both humans and animals and coincidently, is also the same sequence type of the strain harbouring SGI1-B-Ec1 that we recently reported [6].

Additionally, a portion of the backbone of an SGI1-RE within a strain of *Cronobacter* called CFSAN019572 was identified by our analyses. However, a scaffold break thought to be caused by the insertion of an IS*21* family element into *rep* prevented assembly of the complete sequence. The specific aforementioned IS element could not be determined, however, as it was not assembled to a single scaffold. Notably, the integron associated inverted repeats IRi and IRt are not flanked by 5 bp direct repeats, indicating that this locus is unlikely to have resulted from an integration event [19,20] and may instead be a remnant of an earlier homologous recombination event. Preliminary analysis revealed that the structure is flanked by a DR-L and DR-R and appears to have integrated into the terminal 18 bp of the *trmE* gene. It was also noted that the *intI1* gene in this region differed from that of SGI1/PGI2 by 18 SNPs. Only 1 hit within the NCBI nucleotide database (Accession No. LT629801) contained an *intI1* sequence with 100% sequence homology—a strain of *Pseudomonas rhodesiae* which also carried the same chlorine dismutase, *tniC* and *tniQ* genes as the *Cronobacter* integron under analysis, indicating that they share a common origin. An additional six SGI1-RE variants were also identified (Table S1). However, they will not be described in detail due to an inability to determine their complete sequences. Further investigation into these strains, perhaps using long-read sequencing or PCR linkage of SGI1-RE associated scaffolds, would likely reveal novel SGI1-RE variants.

In conclusion, these findings provide insight into the potential evolutionary origins of SGI1 and provide evidence for a greater SGI1 host range than previously recognised. They also highlight that SGI1 and SGI1-REs are part of a large and diverse family of integrative genomic elements whose structures are shaped by a wide range of events including insertion, deletion, and homologous recombination. Once these islands are captured by members of the Gammaproteobacteriaceae, they may come under selection in environments affected by anthropogenic pollutants including antimicrobials, heavy metals, and pharmaceutical residues. As such, it is unsurprising that SGI1-RE can readily acquire multiple antibiotic resistance genes, including genes encoding resistance to clinically important extended-spectrum beta-lactams [2]. These SGI1-REs carrying AMR gene cargo can then disseminate more broadly as they provide their host a fitness advantage in the presence of antimicrobials. Further investigations into the host range of SGI1-REs and more directed searches of these elements within clinically relevant Gram-negative species are needed. More research is also needed to elucidate the phylogenetic relatedness of SGI1-REs. Comparative genomic analysis between SGI1-REs lacking resistance regions, such as those presented in this research and those identified within *Shewanella*, would likely prove informative in this regard [21].

To date, the focus has largely been on the characterisation of SGI1 and SGI1-REs in pathogens. Our studies suggest that further efforts are needed to determine the presence of SGI1-REs in commensal flora circulating in humans, food animals, and the environment more broadly. Additionally, screening for SGI1-RE backbones, rather than phenotypes associated with SGI1-REs, can provide valuable insight into the spread of these elements within Gammaproteobacteriaceae. While the subset of isolates analysed comprises 456,000 genomic datasets uploaded prior to 2017, there are more than 900,000 genomic datasets that are currently available in the SRA. This, however, would require a reindexing of SRA by the tool’s developers. Nonetheless, screening of these additional datasets will likely identify new SGI-REs, enhancing our understanding of the spread of these diverse and clinically significant genomic islands throughout parts of the bacterial Kingdom.

## Supplementary Materials

The following are available online at https://www.mdpi.com/2076-2607/8/2/161/s1, Table S1: Supplementary Table 1.
